# Soziologie des Notfalls

**DOI:** 10.1007/s11577-021-00784-6

**Published:** 2021-07-06

**Authors:** Sebastian Starystach

**Affiliations:** 1 Institut für Medizinische Soziologie und Rehabilitationswissenschaft, Charité Universitätsmedizin Berlin, Charitéplatz 1, 10117 Berlin, Deutschland; 2 Max-Weber-Institut für Soziologie, Ruprecht-Karls-Universität Heidelberg, Bergheimer Straße 58, 69115 Heidelberg, Deutschland

## *Ellebrecht, Nils*:

Organisierte Rettung. Studien zur Soziologie des Notfalls. Wiesbaden: Springer VS 2020. 344 Seiten. ISBN 978-3-658-30161-3. Preis: € 59,99.

Aktuell zeigt uns die COVID-19-Pandemie auf, wie abhängig wir in modernen Gesellschaften von Strukturen organisierter Rettung sind und zugleich in welche Legitimationskrisen diese Systeme und die mit ihr verbunden Maßnahmen geraten können. Vor diesem Hintergrund ist die mit dem Hanno-Peter-Preis der Deutschen Gesellschaft für Katastrophenmedizin e. V. ausgezeichnete Dissertationsschrift Ellebrechts von besonderer Aktualität. Sie gibt umfangreiche soziologische Einblicke in die soziale Konstruktion des Notfalls und seiner organisationalen Bearbeitung.

Zentraler Ausgangspunkt Ellebrechts ist, dass Rettungsorganisationen umfangreich der gesellschaftlichen Erwartung ausgesetzt sind, hoch reliabel Notfallsituationen als solche zu identifizieren, zu klassifizieren und durch Programme erfolgreich zu bearbeiten. Sie genießen ein großes Vertrauen in der Gesellschaft, obwohl es als wahrscheinlich erscheint, dass Notfallorganisationen an sie gerichtete Erwartungen regelmäßig durch erfolglose Rettung auch enttäuschen. Daran anschließend geht die Arbeit zwei zentralen Fragen nach: zum einen, ob Rettungsorganisationen spezifische Strukturmerkmale aufweisen oder ganz normale Organisationen sind und zum anderen, ob das nahezu unerschütterliche Vertrauen, das ihnen gesellschaftlich entgegengebracht wird, auch tatsächlich gerechtfertigt ist. Zur Beantwortung dieser Fragen analysiert Ellebrecht in vier Kapiteln den Notfall als soziale Konstruktion, die Struktur und Funktionsweise von Rettungsorganisationen, die Kooperation zwischen professionellen und hierarchisch-organisierten Rettern und die Triage als notfallmedizinische Rationierung. Dabei greift die Arbeit auf einen systemtheoretischen Zugang nach Luhmann zurück und legt umfangreiches Datenmaterial aus einer Vielzahl unterschiedlich gelagerter qualitativer und quantitativer Studien zugrunde.

Im ersten Kapitel wird ausgehend von einem rechtshistorischen Zugang der Notfall als soziale Situation bestimmt, in der Rettung noch möglich erscheint. Hervorgehoben wird, dass in modernen Gesellschaften der Notfall nicht nur (inter-)subjektiv in der Notfall-Interaktions-Situation erzeugt wird, sondern auch durch Strukturen organisierter Rettung objektiviert wird. Dieser Prozess der Objektivierung ist zwar rechtlich gerahmt, aber nicht rechtlich durchdeterminiert. In der Folge legt auch jede Form organisierter Rettung eine eigene Notfalldefinition zugrunde.

Das zweite Kapitel knüpft an diese Überlegungen an und rekonstruiert, wie Rettungsorganisationen, aber auch mit Rettung betraute Professionen einen objektiven Notfallbezug herstellen. Ellebrecht zeigt nah am Material anhand der Beispiele der Feuerwehr, des Rettungsdiensts, der Polizei sowie Notärztinnen und Notärzten auf, wie durch die organisationale und professionelle Bearbeitung ein lebensweltlich kommunizierter Notfall klassifiziert, entsprechenden Programmen zugeführt und bewältigt wird. Typischerweise prüfen dabei Leitstellen und Wachen misstrauisch anhand ihrer Notfalldefinition, ob der lebensweltlich wahrgenommene Notfall auch ein solcher ist, d. h. in die Zuständigkeit der Rettungsorganisation fällt. Generalisierte Rettungsprogramme klassifizieren dann den Notfall und führen diesen als typisierten Fall einer Bearbeitung zu. Weiterhin zeichnen sich Rettungsorganisationen dadurch aus, dass sie Rettung und Planung schachteln; sie verfolgen damit zwei Zeithorizonte und Ziele: Notfälle können der organisierten Rettung zugeführt werden, sie können aber auch zugleich als Übung für Rettungsprogramme fungieren. Schließlich sind Rettungsorganisationen in der Regel durch ein hohes Maß an Hierarchie und Disziplin geprägt, um ihre Funktion der Rettung hoch reliabel durchführen zu können. Diese Komplexität von Rettungsorganisationen erklärt nach Ellebrecht auch, warum diesen ein umfangreiches Vertrauen geschenkt wird. Aufgrund der Intransparenz der Notfallsituation sind diese umfangreich gegen Kritik geschützt, da sie durch Entscheidungen und die Anwendung professioneller Standards die Situation objektivieren, die dem Laien unverständlich sind. Mit den getroffenen Entscheidungen wird zwar die Verantwortung für die Notfallsituation übernommen, jedoch sind die Entscheidung und ihre Folgen vor dem Hintergrund der dargelegten Komplexität schwer für Dritte kritisierbar.

Das dritte Kapitel beschäftigt sich mit dem Sachverhalt, dass regelmäßig unterschiedliche Rettungsorganisationen und Professionen bei der Bewältigung von Notfällen auf gegenseitige Kooperation angewiesen sind. Ellebrecht zeichnet nach, wie es im Kontext der jeweiligen Objektivierungsleistung zu Widersprüchen kommen kann, wenn unterschiedliche Organisationen und Professionen mit unterschiedlichen Notfalldefinitionen und dazugehörigen Programmen beteiligt sind. Empirisch wird dies diskutiert am Beispiel des latenten Konflikts zwischen dem technisch-naturbeherrschenden Selbstverständnis der hierarchischen Organisation Feuerwehr, die den Notfall als Lage erzeugt und bearbeitet und der professionellen Orientierung von Notärztinnen und Notärzten, die am Patienten als Fall orientiert ist. Die Arbeit zeigt überzeugend auf, dass auch im Kontext moderner Strukturen der Rettung ein Konflikt zwischen professioneller Autonomie und rational-bürokratischer Organisation besteht.

Im vierten Kapitel wird am Beispiel der Triage diskutiert, wie schwer es Rettungsorganisationen fällt, Grenzen ihrer Leistungsfähigkeit anzuerkennen. Die Triage wird vor dem Hintergrund hoher gesellschaftlicher Erwartungen und Professionsethik regelmäßig von Notärztinnen und Notärzten abgelehnt. In der Praxis der Triage zeigt sich, dass Professionsmitglieder sich nicht ohne Weiteres von Algorithmen ihre Entscheidungsautonomie nehmen lassen, sondern selbst im Kontext der Überschreitung der Leistungsfähigkeit, sich an professionell-ethischen und nicht rational-bürokratischen Standards der Leistungszuteilung orientieren.

Insgesamt zeigt die Arbeit nachvollziehbar und empirisch sehr nahe am Untersuchungsgegenstand die Mechanismen und Strukturen organisierter Rettung auf. Es wird überzeugend herausgearbeitet, wie lebensweltliche Situationsdefinitionen organisational objektiviert und damit behandelbar werden. Hier und da ist die Arbeit allerdings schwer für diejenigen nachvollziehbar, die über kein oder nur geringes Vorwissen in Bezug auf Rettungsorganisationen verfügen. Weiterhin fällt auf, dass inhaltlich unterschiedlich gelagerte Studien der Arbeit zugrunde liegen. So sind die Kapitel zwar inhaltlich miteinander verknüpft und durch die oben genannte Fragestellung gerahmt, sie zeichnen sich aber durch heterogene Zugangsweisen (und Schreibstile) aus. Schließlich ist zu bemerken, dass trotz des organisationalen Fokus im Laufe der Arbeit die hervorgehobene Bedeutung der medizinischen Profession im Kontext des Notfalls betont wird. Entsprechende professionssoziologische Überlegungen finden sich zwar dort, wo diese zur Interpretation vorliegender Empirie notwendig sind; eine umfassende und abschließende professionssoziologische Einordnung und theoretische Reflexion dieser Befunde fehlen allerdings.

Insgesamt jedoch sind die Ergebnisse der Arbeit besonders in der aktuellen Pandemiesituation äußerst instruktiv. Denn auch COVID-19 zwingt dazu, aktuelle und potenzielle Grenzen der Leistungsfähigkeit organisierter Rettung zu adressieren. Drohende Triage in Krankenhäusern wird als gesellschaftliches Horrorszenario diskutiert und (politische) Hierarchien und medizinisches Expertenwissen stehen regelmäßig im Konflikt. Es zeigt sich, dass auch heute die soziale Konstruktion des Notfalls umkämpft ist und dies soziologisch nur dann eingeordnet werden kann, wenn in der Analyse berücksichtigt wird, dass unterschiedliche Organisationen und Professionen mit der Bearbeitung derselben Situation unter Zeitdruck und schwindenden Reversibilitätschancen betraut sind. Vor diesem Hintergrund ist dem Buch eine weite Verbreitung zu wünschen.

